# Validation of a short version of the high-fidelity simulation satisfaction scale in nursing students

**DOI:** 10.1186/s12912-023-01515-2

**Published:** 2023-09-28

**Authors:** Alejandro Martínez-Arce, Julián Rodríguez-Almagro, Esperanza Vélez-Vélez, Paloma Rodríguez-Gómez, Alberto Tovar-Reinoso, Antonio Hernández-Martínez

**Affiliations:** 1 Department of Nursing, Fundación Jimenez Díaz School of Nursing- UAM, Madrid, Spain; 2 Department of Nursing, Faculty of Nursing of Ciudad Real, University of Castilla-La Mancha, Ciudad Real, Spain

**Keywords:** Simulation, Undergraduate nursing, Nursing education research, Evaluation

## Abstract

**Background:**

Clinical simulation provides a practical and effective learning method during the undergraduate education of health professions. Currently there is only one validated scale in Spanish to assess nursing students’ satisfaction with the use of high-fidelity simulation, therefore, our objective is to validate a brief version of this scale in undergraduate nursing students with or without clinical experience.

**Method:**

A cross-sectional descriptive study was performed. Between 2018 and 2020, the students from all academic courses of the Fundación Jiménez Díaz nursing school completed the satisfaction scale at the end of their simulation experiences. To validate this scale, composed of 33 items and eight dimensions, exploratory factor analysis (EFA) of the principal components was performed, the internal consistency was studied using Cronbach’s alpha, and the corrected item-test correlation of each of the items of the total scale was reviewed.

**Results:**

425 students completed the scale, after the exploratory factor analysis, a scale consisting of 25 items distributed into six subscales, each containing between two and six items, explained a variance of 66.5%. The KMO test **(**Kaiser-Meyer-Olkin) obtained a value of 0.938, Bartlett’s sphericity test was < 0.01 and Goodness of Fit Index (GFI) was 0.991.

**Conclusion:**

The modified ESSAF scale, reduced from 33 to 25 items and divided into six subscales, is as valid and reliable as the original scale for use in nursing students of different levels, with, or without clinical experience.

**Supplementary Information:**

The online version contains supplementary material available at 10.1186/s12912-023-01515-2.

## Introduction

Some of the new demands that the knowledge society has placed on higher education include encouraging students to develop skills to address situations by applying their knowledge[1]. In the field of nursing education, methodologies aimed at integrating theory with practice are fundamental, assessing both knowledge and skills as well as conveying attitudes [2]. In this context, clinical simulation provides a method of learning and training in which knowledge and skills are intertwined and can lead to learning outcomes that are not achieved through lectures or error with real patients [3].

Simulation has always been present in nursing education, however, in recent years, it has gained significant popularity [4]. Its growth and dissemination are related to the concern for quality and safety in patient care. Specifically, in Spain, simulation is taking center stage in both undergraduate and postgraduate nursing education, with the creation of multiple spaces for simulation within universities, although its implementation and curricular integration is still a challenge [5].

According to Gaba [6], simulation is a technique of learning that amplifies real experiences with guided ones that evoke reality in an interactive manner. It has been shown to be effective for acquiring technical skills and integrating complex clinical knowledge and skills, increasing the degree of retention of what has been learned when compared to traditional teaching methods [7–9]. This type of training is associated with a feedback or debriefing session, in which students and teachers analyze the activity performed, its strengths and areas for improvement, accompanied by a phase of reflective-critical thinking to deepen the trained process [10]. The student assumes an active role in their learning, as the protagonist in the construction of their knowledge in contexts that are similar to reality [11].

Several published meta-analyses have concluded the effectiveness of undergraduate simulation programs vs. traditional teaching models [12]. The meta-analysis published by Cook in 2013 reveals the success factors within simulation programs, highlighting the need for debriefing, integration of simulation into the formal curriculum, individualized simulation practice that is spread over time and demonstrating different variants or clinical contexts [13].

In accordance with these needs, the School of Nursing (Fundation Jiménez Díaz – UAM School of Nursing) has proposed a curricular design where clinical simulation is not an independent subject, but rather it is integrated into the curriculum in a cross-cutting fashion. In the 2018–2019 academic year, simulated clinical experiences were carried out with students in the 1st and 2nd year of the nursing degree within the framework of the subjects Nursing Methodology, Adult Nursing I, II and Psychosociology of Care. In the 2019–2020 academic year, the same simulated clinical experiences were carried out with students in the 1st and 2nd year of the nursing degree and were also carried out in the 3rd and 4th years in the framework of the following subjects: Pediatrics, Psychiatry and Management of Critical Situations. All these simulation activities were designed following the recommendations for a successful simulation program published in the latest systematic reviews [13, 14].

In simulation training, higher student satisfaction results in better learning outcomes, and the design features of a simulation influence its learning outcomes [15], it is essential to increase the impact of the simulated experience by designing simulation scenarios appropriate to the level and learning objectives of the students [16].

In addition, the debriefing that takes place after the simulated event also requires prior preparation and should be related to the completion of the learning process. In our case, each of the simulation modules required at least 3 multidisciplinary work sessions between the teachers responsible for the course, clinical experts and simulation experts, with the aim of designing simulation experiences according to the real needs of the students.

Therefore, it is essential that the teacher receives feedback from the student to understand whether the simulated experience has allowed the student to advance in their learning process or whether it has deviated from their real needs in order to complement their theoretical knowledge base [17].

According to the Standards of Best Practice in simulation [18], teachers should ensure the effectiveness of the overall experience with the goal of identifying aspects of the simulation program that support optimal transfer of knowledge, skills and overall competence into practice. This evaluation of the simulation program should be comprehensive, combining evaluation of activities before, during and after the simulations [19].

In this regard, several instruments have been developed to measure student satisfaction in the field of clinical simulation, teamwork and decision making, among others [20–23]. At present, the only scale validated in Spanish is the High-Fidelity Simulation Satisfaction Scale for Students (ESSAF). This is a 33-item questionnaire, validated by Alconero et al. [12], which assesses student satisfaction and evaluates the students’ perception of the usefulness of clinical simulation training, together with other aspects. This questionnaire was validated with an initial sample of 150 students from the same academic year and we do not know if it is valid for use with students with different experience, since the evidence determines the importance of adapting the type of simulation design most appropriate for the experience of the student [24].

Although this is a valid questionnaire, it is conformed of 33 items, which makes it extensive, and therefore difficult to systematically implement for evaluating satisfaction of all simulations. The development of a simplified version with the same psychometric characteristics would be a way to improve the universalization of this evaluation system. For this reason, the aim of this study was to validate a brief version of the ESSAF questionnaire for its application in the different academic years of the nursing degree and in students with or without clinical experience.

## Materials and methods

### Design

A cross-sectional descriptive study was employed within the framework of a Teaching innovation project funded by Universidad Autónoma de Madrid (UAM) of undergraduate Nursing students belonging to the Fundaci?n Jimónez Díaz - UAM School of Nursing. The study population was 1st and 2nd year students of the 2018–2019 academic year and 1st, 2nd, 3rd, and 4th year students of the 2019–2020 academic year. 425 students completed the satisfaction survey.

During the months of May to July 2018, the initial simulation program was designed by a multidisciplinary working group over a series of six work sessions. The decision was made to start with first and second year students in order to consolidate and apply the theoretical knowledge prior to their clinical practices, extending it to all courses during the following academic year.

### Sample selection

The criteria for carrying out a factorial analysis were used to calculate the sample size. These criteria contemplate 10 subjects for each [25]; therefore, a sample of at least 330 participants was needed.

### Description of the activity

A total of 32 simulation sessions were carried out throughout the 2018–2019 academic year, and a total of 59 sessions took place during the 2019–2020 academic year. In each simulation session, small groups of 8–10 students participated, with an approximate duration of two to four hours, in which three scenarios were developed. These sessions were recorded on a video system and viewed in real time by the students. The following link shows an example of a scenario carried out by third year students for verbal restraint of a psychiatric patient. https://www.youtube.com/watch?v=b8gM5u2ihsA.

All simulation scenarios were performed with the same teaching design:

1. Prebriefing or introduction to clinical simulation.
2. Patient presentation and work environment.
3. Three simulated clinical scenarios in which all trainees participated in at least one of the scenarios.
4. A debriefing following each of the scenarios using the sound judgment approach [10].

To conduct the simulation, a main instructor was in charge of clinical simulation immersion and of coordinating the debriefing. In addition, a co-instructor provided support as expert staff of the subject to be trained, and for the management of the simulator and video recording systems. On occasions, actors were needed to faithfully recreate the real situation.

### Data collection

The ESSAF scale (Additional File 1) was used, which is a self-administered questionnaire that students completed voluntarily and anonymously at the end of the simulation module. This scale contains 33 statements answered on a 5-point Likert-type scale, with a minimum score of 1 (strongly disagree) to 5 (strongly agree). With appropriate indicators for factoring, the 33 items are grouped into 8 factors or dimensions of student perception of clinical simulation: “Usefulness”, “Characteristics of cases and applications”, “Communication”, “Perceived performance”, “Increased self-confidence”, “Relationship between theory and practice”, “Facilities and equipment” and “Negative aspects”.

Sociodemographic variables such as students’ age, sex and academic year were also collected.

To facilitate data collection and guarantee anonymity, an ad-hoc questionnaire was generated and completed by the students at the end of the simulation practices (Additional File 1. Supplementary material: ESSAF Questionnaire). All students who completed the simulation sessions were included, excluding those who for any reason did not complete the sessions.

### Data analysis

All questionnaires were numerically coded using SPSS version 20 statistical software for data collection and analysis.

In terms of baseline quality control, all variables included in the study were analyzed for missing values or errors in data recording (analysis for out-of-range values, incomplete data, statistical analysis for errors or outliers (descriptive, frequencies, means, range).

The distribution of variables was determined by descriptive analysis of the variables and the use of Q-Q graphs, histograms, and box plots; in the event of doubt, the Kolmogorv-Smirnov statistical test was used, in which the null hypothesis assumes normal or Gaussian distribution of the variable. For variables with normal distribution, parametric analyses were used. If the variables had a non-Gaussian distribution, nonparametric analyses were used.

Descriptive statistics: the results of quantitative variables with a normal distribution were expressed by their mean and standard deviation (SD). Quantitative variables with a non-Gaussian distribution were expressed as median and interquartile range (IQR), and qualitative variables were expressed as frequency and percentage. Ordinal variables were analyzed as continuous variables, expressed as median and interquartile ranges. To facilitate comprehensive data interpretation for our readers, both mean (SD) and median (IQR) values will be provided. This approach ensures clarity, especially when certain data exhibit a normal distribution while others do not.

In the present study, the option recommended by several authors was used [26, 27], exploratory factor analysis (EFA) based on polychoric correlations, given that in the univariate analysis of the ordinal items the authors find an excess of kurtosis and skewness. The robust unweighted least squares (ULS) method was used as the factor estimation method [26]. Parallel analysis (PA) was used as the factor selection retention method, and the PROMIN method was used as the factor rotation method.

The FACTOR program (version 10.9.02) was used for Exploratory Factor Analysis (EFA). Initially, a descriptive analysis was conducted for each item, assessing mean, standard deviation, skewness, and the corrected item-test correlation. To minimize noise in the subsequent factor analysis, items were removed with correlations below 0.20, as recommended [27]. We further scrutinized the distribution of items by evaluating the kurtosis and skewness coefficients [28]. Following Kline’s criteria [29], item-test was corrected correlation for the entire scale, excluding items with values below 0.20.

To gauge reliability, which we define as the internal consistency of items measuring a construct, we relied on both the ORION coefficient and Cronbach’s Alpha. ORION (an acronym for “Overall Reliability of fully Informative prior Oblique N-EAP scores”) measures the overall reliability of the aforementioned oblique scores [30]. While the Cronbach’s Alpha coefficient is grounded on the mean correlations between items, it remains the most popular statistic for internal consistency, despite some controversies surrounding it [31–33].

### Ethical considerations

The study was sent for evaluation to the ethics committee of the Universidad Autónoma de Madrid, which ruled that the project did not contradict ethical standards and did not need to be evaluated as it was a satisfaction survey.

All experimental protocols were approved by the ethics committee of the Universidad Autónoma de Madrid on October 1, 2021.

Participants were informed of the study and confirmed their informed consent to participate in the research. All data were treated confidentially in accordance with the Organic Law 3/2018 of 5 December on the Protection of Personal Data and Guarantee of Digital Rights, keeping them strictly confidential and non-accessible to unauthorized third parties and the Regulation (EU) 2016/679 of the European Parliament and of the Council of 27 April 2016 on Data Protection (GDPR). The simulation scenarios were recorded for later analysis during the debriefing, all participants were informed that the recordings would be used exclusively for teaching or research use and signed their informed consent to the recording.

## Results

### Sociodemographic characteristics

Of the total number of students who completed the satisfaction survey (N = 425), 88.0% (374) were female and the mean age was 20.11 years (SD = 5.34 years) with a median of 19 years (min and max: 18–49), respectively. First year students accounted for 34.5% (147), second year students accounted for 29.8% (127), third year students accounted for 16.7% (71) and fourth year students accounted for 18.8% (80). This more detailed information can be found in Table 1.

**Table 1:** Sample characteristics

Variables		Total n = 425 n (%)
**Sex**		
	Women	374 (88.0%)
	Men	51 (12.0%)
**Age**		
	18–20 years	278 (65.4%)
	21–23 years	100 (23.5%)
	24–30 years	19 (4.5%)
	Over 30 years	16 (3.8%)
**Experience with simulation**		
	No	298 (70.1)
	Yes	127 (29.9)
**Academic year**		
	First year	147 (34.6)
	Second year	127 (29.9)
	Third year	71 (16.7)
	Fourth year	80 (18.8)

Number and percentage of students by age, gender, and degree of experience

### Exploratory factor analysis

The KMO test **(**Kaiser-Meyer-Olkin) obtained a value of 0.938, and Bartlett’s sphericity test was < 0.01. Therefore, we proceeded to carry out the EFA. Six components explained 66.5% of the variance. To determine the number of factors, the parallel analysis suggested the existence of three factors, however, the starting theory in the development of the questionnaire and the interpretation of the solution found were decisive in obtaining the six factors shown, and the estimation method was unweighted least squares with promin rotation [34], chosen due to the extreme distribution of the data.

The first model of the analysis shows items with saturations lower than 0.3 in any of the six factors considered, and in these cases the items were removed one by one, repeating the analysis each time. Pursuing the principle of parsimony, in order to obtain the simplest model and the most easy to interpret, the items or complex variables were also removed, those that have little influence on the definition of the factor and also do not show a clear belonging to only one factor. Table 2 shows the factor loadings of the modified ESSAF scale and correlation matrix.

**Table 2:** Factor loadings of the modified ESSAF-scale and correlation matrix

N = 425	F1	F2	F3	F4	F5	F6
V9. Simulation will help me to establish priorities for action in clinical situations.	**0.960**	0.050	-0.304	-0.303	0.030	-0.235
V15. Simulation enables effective patient care planning	**0.739**	-0.038	0.103	0.007	-0.035	0.101
V19. This experience will help me to prioritize care	**0.714**	0.078	-0.091	-0.064	-0.098	0.021
V10. Simulation will improve my ability to provide care to my patients.	**0.648**	0.014	-0.142	-0.042	0.285	-0.084
V14. Simulation is beneficial because it relates theory to practice.	**0.635**	0.125	0.189	0.032	-0.085	0.109
V16. Simulation will improve my technical skills	**0.563**	-0.155	0.013	0.201	0.263	-0.127
V29. Debriefing helps to correct mistakes	0.078	**0.834**	0.072	0.150	0.117	-0.123
V28. Debriefing allows for reflection on cases	0.265	**0.740**	0.382	-0.035	-0.036	0.003
V27. The teacher always gives constructive feedback after each simulation session	-0.341	**0.529**	0.044	-0.058	0.405	-0.085
V2. The objectives of the simulation of the cases are clear	-0.126	0.132	**0.566**	-0.138	0.406	-0.100
V1. The simulation classrooms where the cases take place are real	-0.103	-0.033	**0.564**	-0,007	0.172	-0.143
V7. Simulation is useful for assessing a patient’s clinical situation	0.218	0.002	**0.445**	0.062	0.107	-0.164
V18. Simulation will help me to assess the patient’s condition	0.300	-0.117	**0.436**	0.241	-0.002	0.065
V12. Simulation will improve the ability to work with the equipment	-0.215	0.141	-0.101	**0.998**	0.155	0.111
V17. The simulation will reinforce my critical thinking and decision making	0.325	-0.200	0.091	**0.621**	0.053	-0.125
V21- Simulation improves communication with the team	0.076	0.135	0.018	**0,557**	0.088	0.025
V11. The simulation will make me reflect on my next clinical practice	0.275	-0.003	-0.085	**0.481**	0.166	-0.198
V31. Simulation will allow me to learn from the mistakes I have made	0.089	0.108	-0.061	− 0.201	**0.864**	0.003
V24. Simulation will increase my safety	-0.230	-0.006	-0.022	0.189	**0.639**	0.019
V32. Simulation is useful in practice	0.330	-0.058	-0.007	-0.059	**0.628**	0.033
V20. Simulation promotes self-confidence	-0.116	-0.086	0.119	0.181	**0.601**	0.016
V26. Simulation will improve my clinical competence	0.167	-0.003	-0.073	**-**0.029	**0.595**	0.137
V33. With these sessions I will meet the expected learning outcomes	0.172	0.053	0.058	0.062	**0.490**	0.055
V23. Improved communication with the patient	-0.077	0.052	-0.067	0.195	0.118	**0.710**
V22. Improved communication with the family	0.083	-0.072	0.032	0.085	-0.015	**0.643**
Matrix determinant = 0.000002517365237 Bartlett’s Test of Sphericity = 5361.0 (df = 300; p < 0.001) Kaiser-Meyer-Olkin (KMO) test = 0.93810 (very good) % Explained variance = 66.52% **Goodness-of-fit indicators**: • Root Mean Square Error of Approximation (RMSEA) = 0.023; Good fit if < 0.05 • Non-Normed Fit Index (NNFI; Tucker & Lewis) = 0.996 • Comparative Fit Index (CFI) = 0.998; (> 0.990: excellent) • Goodness of Fit Index (GFI) = 0.991 • Adjusted Goodness of Fit Index (AGFI) = 0.9678 Root Mean Square of Residuals (RMSR) = 0.0596; Expected mean value of RMSR for an acceptable model = 0.0836 (Kelley’s criterion) [33].						

### Denomination of the factors

Considering the items grouped in each factor and the weights of each one of them, the six factors have been named as shown in Table 3. These six factors encompass the entire clinical simulation training process in students, evaluating the benefits or impact of the methodology in aspects of previous planning, (F3) direct care in various dimensions such as care, (F1) teamwork and critical reasoning, (F4) learning, safety, and confidence (F5) and communication with the patient and family (F6) and finally, in the subsequent feedback or debriefing (F2).

**Table 3:** Reliability of the factors, and replicability index

Factors	ORION Reliability	Cronbrach’s Alpha (α)	G- H* Index
F1: Impact of simulation on care (6)	0.918	0.758	0.902
F2: Benefits of feedback in simulation (3)	0.903	0.701	0.915
F3: Benefits or usefulness of pre-planning (objectives, infrastructure) (4)	0.772	0.612	0.846
F4. Benefits on teamwork and critical thinking (4)	0.914	0.759	0.910
F5. Benefits on learning, safety and confidence	0.924	0.812	0.870
F6. Benefits on communication with patient and family	0.818	0.709	0.810

* The H-index evaluates how well a set of elements represents a common factor. It is bounded between 0 and 1 and approaches unity as the magnitude of factor loadings and/or the number of items increases. High H values (>0.80) suggest a well-defined latent variable, which is more likely to be stable across studies, while low H values suggest a poorly defined latent variable, which is likely to change across studies [34].

### Reliability and replicability of the data collection tool

Table 3 presents the reliability and replicability indices of the modified ESSAF- Scale. With either index, factor reliability was good (≥ 0.70), with factor 3 having the lowest or borderline reliability. Moreover, the replicability of the scale factors is shown with the H-incident that evaluates how well a set of items or elements represents a common factor (values > 0.80 suggest a well-defined latent variable, which is more likely to be stable between different studies), observing that all of them have good replicability, with values close to unity.

### Applicability

Table 4 shows the descriptive analysis of the scale reduced to 25 items, as well as the number of items in each subscale, with the mean and median scores obtained in our sample. Additional File 2 includes a table with the definitive distribution of the items for each of the subscales.

**Table 4:** Descriptive analysis of ESSAF- adapted

ESSAF subscales n = 425	Possible scores	No. of Items	Mean (SD)	Median (IQR)
Impact of simulation on care	6–30	6	28.10 (2.11)	29 (27–30)
Benefits of feedback in simulation	3–15	3	14.71 (0.73)	15 (15–15)
Benefits of pre-planning	4–20	4	18.67 (1.44)	18 (19–20)
Benefits on teamwork and critical thinking	4–20	4	18.65 (1.72)	18 (19–20)
Benefits on learning, safety and confidence	6–30	6	28.02 (2.25)	27 (29–30)
Benefits on patient and family communication	2–10	2	8.70 (1.39)	9 (8–10)

SD: Standard deviation; IQR: Interquartile range

## Discussion

The ESSAF scale reduced to 25 items and 6 factors, assessing pre-, during and post-simulation (debriefing) aspects with high reliability, makes this scale a simpler and more reliable tool than the original one, which will facilitate comprehensive simulation program evaluation.

This need for comprehensive simulation program evaluation has increased as a result of the development of best practice standards and is a key point for academic and clinical simulation programs to determine if efforts to improve knowledge, skills and/or attitudes have been effective [18, 19]. At the same time, this assessment can be complex and having a simple tool that is applicable to students with different academic backgrounds can help in this evaluation process.

The ESSAF scale presented good internal reliability (α = 0.859) and high replicability indices (H-index close to unity). However, the reliability analysis of the different dimensions in the present study does not replicate the good reliability found by its authors [12]; only two of the 8 factors of the ESSAF tool presented an α ≥ 0.70. The availability of a larger sample, with students from different academic years has made it possible to simplify the sample and to eliminate items, as well as a new classification by subscales or factors.

These six factors perfectly cover all the key aspects of simulation training [35] and encompass all areas of training described in the literature in a simple and reliable manners for all levels of experience in the nursing curriculum. Not only are they focused on the direct assessment of nursing care, but the factors enable the assessment of the benefit on cognitive competencies of reasoning and prior preparation or the benefit of feedback or subsequent debriefing which is currently considered the key to any clinical simulation activity [13, 35] and it is not always evaluated as reflected in the systematic review by Levett-Jones and Lapkin (2014) who included 10 controlled studies in undergraduate nursing, only two of which addressed the benefit or impact of feedback or subsequent debriefing [36].

Table 4 shows the mean scores of the different factors and, as mentioned in the literature reviewed, the factor referring to debriefing (F2) has the highest scores of all the factors, which reflects that our students have recognized the importance of debriefing to generate new models of thinking and how to apply them in future practice [10].

As for the factors that encompass direct care competencies, we can perfectly differentiate those that are not only focused on care (F1) and that are becoming increasingly important in the curricular design of undergraduate training, such as communication skills with patients and family (F6), teamwork (F4) and safety and confidence (F5), all of which were recently highlighted in a systematic review showing the usefulness of simulation training for acquiring these types of competencies [37].

Finally, the factor related to the benefits or usefulness of pre-planning (F3) is going to allow to complete that comprehensive evaluation described in the literature, in this case focused on the pre-simulation part and that will help in the narrative of the clinical scenario, which is related to the decision making in the scenario and also the level of complexity and how to adjust the information given to the students. The teacher can use this to guide decisions on the types of information to provide to adjust the complexity of the clinical scenario [11, 38].

### Limitations

As for the limitations of this work, we began with a very limited sample chosen by non-probabilistic convenience sampling. Although various systems are currently used to determine the number of subjects required for validation studies, such as the N/p type criterion and the criterion of 10 times more subjects than items, among others, they are completely discouraged, as they have no solid basis [27]. In fact, there is no consensus, since the minimum recommended size depends on numerous factors. Logically, the larger the sample size available, the more confidence we will have that the solution obtained is stable, especially if the communality is low, or when we have many possible factors to extract and/or few items per factor. Nonetheless, to evaluate the quality of a test, clearly, a sample size of at least 200 cases is recommended, even under optimal conditions of high communalities and well-determined factors [27]. We opted for the criterion of 10 subjects per item, which represents a sample much larger than 200 and therefore adequate for the purpose of the study.

Another of the limitations observed can be found in the homogeneity of the sample where there is an imbalance between the characteristics of the participants since 70.1% have no previous experience in simulation and there is also a higher percentage of first and second year students. However, we consider that it could be a strength because the psychometric characteristics are adequate despite being a non-homogeneous sample.

## Conclusions

Conducting ongoing evaluation of the simulation program provides teachers with the data needed to recognize and implement changes in future simulation experiences.

We have observed that the modified ESSAF scale, divided into six subscales, is a practical and reliable tool to be used by nursing students from different academic years and with different degrees of clinical experience, compared to the original scale. This new classification is very useful to provide teachers with feedback not only in relation to the competencies acquired, but also in relation to the design of the simulated clinical experiences and their subsequent analysis or debriefing.

Evaluating each simulation program with different tools can be complex and tiring for teachers and students. This simple and concise tool can be the first step in evaluating a simulation program for nursing students in a comprehensive manner and guide a second, more concise evaluation phase on relevant aspects that have been detected.

### Electronic supplementary material

Below is the link to the electronic supplementary material.

Supplementary Material 1

Supplementary Material 2

## Data Availability

The datasets used and/or analyzed during the current study are available from the corresponding author on reasonable request.

## Abbreviations

EFA: Exploratory factor analysis
EU: European Union
ESSAF: High-Fidelity Simulation Satisfaction Scale for Students
GDPR: General Data Protection Regulation
GFI: Goodness of Fit Index
IQR: Interquartile range
KMO: Kaiser-Meyer-Olkin
ORION: Overall Reliability of fully Informative prior Oblique N-EAP scores
PA: Parallel analysis
SD: Standard deviation
SPSS: Statistical Package for the Social Sciences
ULS: Unweighted least squares
