# Supplementary material for: Validation of a short version of the high-fidelity simulation satisfaction scale in nursing students
Source: BMC Nurs. 2023 Sep 28;22:344. doi: 10.1186/s12912-023-01515-2 (PMC10537079; doi:10.1186/s12912-023-01515-2)
Supplement: Supplementary file 1 — Supplementary Material 1 [file 12912_2023_1515_MOESM1_ESM.docx]

**Annex 1: Original scale**

| **Items** | **Degree of agreement** | | | | | | | | | |
| --- | --- | --- | --- | --- | --- | --- | --- | --- | --- | --- |
|  | **Strongly Strongly**  **Desagree Agree** | | | | | | | | | |
| 1. Do you think that the simulation classrooms where the cases are developed are real? | 1 | 2 | | 3 | | 4 | | | 5 | |
| 2. Are the objectives of the simulation cases clear? | 1 | 2 | | 3 | | 4 | | | 5 | |
| 3. Do the cases recreate real situations? | 1 | 2 | | 3 | | 4 | | | 5 | |
| 4. Is the time dedicated to simulation sessions adequate? | 1 | 2 | | 3 | | 4 | | | 5 | |
| 5. Is the degree of difficulty of the cases adequate to my knowledge? | 1 | 2 | | 3 | | 4 | | | 5 | |
| 6. I felt comfortable and respected during the sessions | 1 | 2 | | 3 | | 4 | | | 5 | |
| 7. The simulation is useful to evaluate the clinical situation of a patient | 1 | 2 | | 3 | | 4 | | | 5 | |
| 8. By practicing in simulation you learn not to make mistakes | 1 | 2 | | 3 | | 4 | | | 5 | |
| 9. Simulation will help me to establish priorities of action in clinical situations | 1 | 2 | | 3 | | 4 | | | 5 | |
| 10. The simulation will improve my ability to provide care to my patients | 1 | 2 | | 3 | | 4 | | | 5 | |
| 11. The simulation will make me think about my next clinical practice | 1 | 2 | | 3 | | 4 | | | 5 | |
| 12. Simulation will improve my communication and ability to work with the team | 1 | 2 | | 3 | | 4 | | | 5 | |
| 13. The simulation will increase my level of concern regarding the competencies that a registered nurse must have | 1 | 2 | | 3 | | 4 | | | 5 | |
| 14. Simulation is beneficial in that it relates theory to practice | 1 | 2 | | 3 | | 4 | | | 5 | |
| 15. Simulation allows for effective planning of patient care | 1 | 2 | | 3 | | 4 | | | 5 | |
| 16. Simulation will improve my technical skills | 1 | 2 | | 3 | | 4 | | | 5 | |
| 17. Simulation will reinforce my critical thinking and decision making | 1 | 2 | 3 | | 4 | | | 5 | | |
| 18 Simulation will help me to assess the patient's condition | 1 | 2 | 3 | | 4 | | | 5 | | |
| 19. The experience will help me to prioritize care | 1 | 2 | 3 | | 4 | | | 5 | | |
| 20. Simulation will promote self-confidence | 1 | 2 | 3 | | 4 | | | 5 | | |
| 21. Simulation enhances communication with the team | 1 | 2 | 3 | | 4 | | | 5 | | |
| 22. Simulation improves communication with the family | 1 | 2 | 3 | | 4 | | | 5 | | |
| 23. Simulation improves communication with the patient | 1 | 2 | 3 | | 4 | | | 5 | | |
| 24. Simulation will increase my safety | 1 | 2 | 3 | | 4 | | | 5 | | |
| 25. During the simulation sessions, you may "lose your temper" with some of the cases | 1 | 2 | 3 | | 4 | | | 5 | | |
| 26. Simulation will improve my clinical competency | 1 | 2 | 3 | | 4 | | | 5 | | |
| 27. The professor always gives constructive feedback after each simulation session | 1 | 2 | 3 | | 4 | | | 5 | | |
| 28. The analysis (debriefing) allows me to reflect on the cases | 1 | 2 | 3 | | 4 | | | 5 | | |
| 29. The debriefing helps to correct errors | 1 | 2 | 3 | | 4 | | | 5 | | |
| 30. I know the theoretical part of the cases beforehand | 1 | 2 | | 3 | | | 4 | | | 5 |
| 31. The simulation will allow me to learn from the mistakes I made | 1 | 2 | | 3 | | | 4 | | | 5 |
| 32. Simulation is useful in practice | 1 | 2 | | 3 | | | 4 | | | 5 |
| 33. With the development of these sessions I will meet the learning expectations | 1 | 2 | | 3 | | | 4 | | | 5 |
